# Cytopathic and Genomic Characteristics of a Human-Originated Pseudorabies Virus

**DOI:** 10.3390/v15010170

**Published:** 2023-01-05

**Authors:** Zhong Peng, Qingyun Liu, Yibo Zhang, Bin Wu, Huanchun Chen, Xiangru Wang

**Affiliations:** 1 State Key Laboratory of Agricultural Microbiology, College of Veterinary Medicine, Huazhong Agricultural University, Wuhan 430070, China; 2 Key Laboratory of Preventive Veterinary Medicine in Hubei Province, The Cooperative Innovation Center for Sustainable Pig Production, Huazhong Agricultural University, Wuhan 430070, China; 3 Key Laboratory of Development of Veterinary Diagnostic Products, Ministry of Agriculture of China, Huazhong Agricultural University, Wuhan 430070, China; 4 International Research Center for Animal Disease, Ministry of Science and Technology of China, Wuhan 430070, China

**Keywords:** pseudorabies virus, human, pig, complete genome sequence, comparative genomics

## Abstract

Pseudorabies virus (PRV) generally infects pigs and threatens the pig industry. However, recently we have isolated a PRV strain designated hSD-1/2019 from infected humans. In this study, we compared the complete genome sequence of hSD-1/2019 with those of pig-originated PRV strains. Sequence alignments revealed that the genome sequence of hSD-1/2019 was highly homologous to those of the porcine PRV strains. Phylogenetic analyses found that hSD-1/2019 was the closest related to porcine PRV endemic strains in China, particularly the variant strains circulating recently. We also showed that the glycoproteins important for the multiplication and pathogenesis of hSD-1/2019 were highly similar to those of the pig endemic strains. Diversifying selection analyses revealed that hSD-1/2019 and pig variant strains are under diversifying selection. Recombination analysis indicated that hSD-1/2019 was a recombinant of several PRV variant strains and an earlier PRV classic strain. Finally, we found that both human and pig-originated PRV strains could induce cytopathic effects in cells from humans, pigs, and mice, but only the human PRV and pig-variant PRV formed large syncytia in human cell lines. The data presented in this study contribute to our understanding of the molecular basis for the pathogenesis of human PRV from a genomic aspect.

## 1. Introduction

The world is now under the One Health Initiative, in which there is no dividing line between human and animal medicine [1]. Indeed, approximately 61% of the infectious organisms affecting humans are zoonotic [2]. The epidemic or pandemic of wildlife-origin pathogens, including Ebola and Marburg virus, human immunodeficiency virus (HIV)-1 and HIV-2, Sin Nombre virus, Nipah, Hendra and Menangle virus, West Nile virus, Borrelia burgdorferi, SARS coronavirus, MERS coronavirus, and more recently, SARS-CoV-2, have caused huge morbidity, mortality, and economic loss for humans. However, much of the knowledge about the pathogenesis and interspecies transmission of these pathogens is poorly understood.

Pseudorabies virus (PRV) is a double-stranded DNA virus belonging to the genus *Varicellovirus* of the subfamily *Alphaherpesvirinae*, family *Herpesviridae* [3]. PRV generally possesses a linear DNA genome with high G+C content (approximately 74%); this 143-kb genome encodes 70~100 proteins involved in the formation of viral capsid, tegument, and envelope. Among these proteins, glycoproteins gB, gD, gH, gL, and gK are necessary for virus multiplication, while gE, gL, gG, gC, gM, and gN are the main virulence determinants [4]. It is worth noting that gC protein has also been used as a marker for PRV genotyping, and based on this gene, PRV strains are divided into two genotypes: genotype I and genotype II [5]. In general, PRV strains cause lethal infections in many animal species, with the exception of pigs, and reproductive failure in sows, as well as respiratory and neurological symptoms in piglets, are the common manifestations in pigs [6,7]. In China, the first report of a PRV outbreak in pigs occurred in the 1950s, and an inactivated vaccine consisting of PRV strain Bartha was imported into China in the 1970s [6]. Between 1990 and 2011, the wide vaccination of this inactivated vaccine in pig herds contributed to the control of PRV outbreaks well in China [8]. However, in late 2011, PRV variant strains emerged and circulated in many Bartha-K61-vaccinated pig farms in China [9,10]. These viruses display higher pathogenicity than PRV strains circulating in China before, and they show a different genotype from the Bartha strain [6].

For a very long time, whether humans are susceptible to PRV infection has been the subject of controversy, although several pieces of serological evidence have been found [7]. However, 25 cases of suspected PRV infection in humans were reported in China between 2017 and 2021, and all of the infected individuals in these cases had a history of working near pigs or in pork production [11,12,13,14,15,16,17,18]. These reported cases suggested that PRV infection might represent a new threat to humans in China. While in most of these cases, PRV-specific sequences have been determined in patients’ tissues, none of them have reported the successful isolation of PRV. Recently, our group reported the isolation of the first PRV strain from the cerebrospinal fluid (CSF) of an infected patient with acute encephalitis in China [18]. To further explore the genetic characteristics of this human-originated PRV strain and its association with the pig PRV strains, we performed a comparative genomic analysis of the pseudorabies virus originating from humans and pigs in this study. Our aim is to provide more knowledge about the pathogenesis and interspecies transmission of PRV from a genomic perspective.

## 2. Materials and Methods

### 2.1. PRV Strains, Cells, Culture Conditions, and Whole-Genome Sequences

The PRV strains used in this study included hSD-1/2019 (GenBank accession no. MT468550), HuBXY/2018 (GenBank accession no. MT468549), and Ea (GenBank accession no. KX423960). These three PRV strains are all clinical isolates preserved in our laboratory: hSD-1/2019 was isolated from the CSF of an infected veterinarian with acute encephalitis in a pig farm of Shandong Province in China 2019 [18]; HuBXY/2018 is a variant isolated from the brain tissue of a piglet with neurological symptoms in Hubei Province in 2018; and Ea is a classic PRV epidemic strain isolated from pigs in China in the 1990s. The detailed steps for the isolation of hSD-1/2019 using PK-15 cells (ATCC, CCL-33) have been documented in our recent publication [18].

Cell lines, including PK-15 (Porcine Kidney-15; ATCC, CCL-33), ST (Swine Testis Cells; ATCC, CRL-1746), HT-22 (Mouse Hippocampal neuronal cell line; Sigma-Aldrich, SCC129), ARPE-19 (Adult Retinal Pigment Epithelial cell line-19; ATCC, CRL-2302), hBMEC (Human Brain Microvascular Endothelial Cells; gifted by Prof. Kwang Sik Kim at Johns Hopkins University School of Medicine), SK-N-SH (human neuroblastoma cell; ATCC, HTB-11), and hUVEC (Human Umbilical Vein Endothelial Cells; ATCC, CRL-1730) were used in this study. Among these cells, the PK-15, ST, HT-22, and ARPE-19 cells were maintained in Dulbecco’s Modified Eagle Medium (DMEM; Gibco, Thermo Fisher Scientific, Waltham, MA, USA) supplemented with 10% fetal bovine serum (Genimi Bio, Calabasas, CA, USA); the hBMEC cells were cultured using RPMI 1640 medium (Gibco, Thermo Fisher Scientific, Waltham, MA, USA) supplemented with 10% fetal bovine serum; the SK-N-SH cells were maintained in Minimum Essential Medium (MEM; Gibco, Thermo Fisher Scientific, Waltham, MA, USA) supplemented with 10% fetal bovine serum.

The whole-genome sequences used for the analyses in this study included those of 55 pig epidemic strains that we isolated in China between 2011 and 2018 [6], as well as those of several other pig-originated epidemic strains in China. The genome sequences of all of these PRV strains were retrieved from NCBI, and their GenBank accession numbers are listed in Table S1 in the Supplementary Materials.

### 2.2. Genomic DNA Extraction and Pacific Biosciences (PacBio) Sequencing

To obtain high-quality genomic DNA for PacBio sequencing, the isolated human PRV strain, hSD-1/2019, was passaged using PK-15 cells for 5 generations and was then inoculated in PK-15 cells at 0.1 MOI. A total of 100 mL of the viral culture was prepared. The viral culture was centrifuged together with a sucrose solution (30% *m/v*) at 26,000 rpm for 3 h; then the viral pellets were washed and resuspended in PBS. The genomic DNA was extracted using the phenol–chloroform protocol, as described previously [19]. The DNA concentration, quality, and integrity were evaluated using a Qubit Flurometer (Invitrogen, Thermo Fisher Scientific, Waltham, MA, USA) and a NanoDrop Spectrophotometer (Thermo Scientific, Waltham, MA, USA). Afterward, a TruSeq DNA Sample Preparation Kit (Illumina, San Diego, CA, USA) and a Template Prep Kit (Pacific Biosciences, CA, USA) were used for the preparation of the 20-kb sequencing libraries, which were then sequenced on the Pacific Bio sciences platform and the Illumina Miseq platform at Personal Biotechnology Company (Shanghai, China). This sequencing strategy yielded 324,157,231-bp raw reads (*N*_50_, 13277 bp). In the next step, the adapter contaminations were removed and the data were filtered by using AdapterRemoval [20] and SOAPdenovo2 [21]. Through this approach, a total of 7,708,624-bp clean reads (Q20% > 96.63%; Q30% > 88.84%) were obtained for de novo assembly using SPAdes [22] and A5-miseq [23] to construct the scaffolds and contigs. The Canu v1.5 [24] package was used to assemble the data obtained through the PacBio sequencing. All of the assembled data were integrated to generate a complete sequence. The final genome sequence was acquired after the rectification by using pilon software [25].

### 2.3. Bioinformatic Analysis

The sequence alignments were performed by using the EasyFig package (version 2.2.3_win) [26] and/or the MAFFT package (version 7.471) [27]. The nucleotide similarities at the genome level were calculated and visualized by using the SimPlot software (version 3.5.1), and the data were regenerated by using GraphPad Prism 8. GeneDoc (version 2.7) was used to visualize the sequence alignments of the genes or proteins. The average nucleotide identity (ANI) between the two genome sequences was calculated by using the ANI calculator [28]. Phylogenetic analysis was performed using the Beast 2 program (version 2.6.3) [29]. By using this program, maximum likelihood trees were generated through the Gamma correction for site heterogeneity and the GTR model [30]. A bootstrap value of 1000 was also applied, and the tree was visualized by using the iTOL tool [31]. The single-nucleotide polymorphisms (SNPs) between the different PRV strains were determined by using the MUMmer software (version 3.23) [32]. The coding effects of the SNPs were determined by using a local Perl command described previously [33]. The recombinant sequences were determined by using the Recombination Detection Program (RDP) package Beta 4.100 [34]. A recombination event with a significance of *p* < 0.01 in at least three out of seven of the selected algorithms: RDP, GENECONV, BootScan, Maxchi, Chimaera, SiScan, and 3Seq, was considered to be reliable, as previously described [35].

### 2.4. Cells Infection Tests

The cell monolayers were infected with PRV hSD-1/2019 (human-originated strain), HuBXY/2018 (pig-originated clinical variant strain), or Ea (pig-originated clinical classic strain) at 5 MOI and were incubated at 37 °C. At 12 h post-infection, the cytopathic effects were observed and recorded using the EVOS^®^ FL Auto Imaging System (Thermo Fisher Scientific, Waltham, MA, USA).

## 3. Results

### 3.1. Overview of the Human-Originated PRV Genome

**Figure 1:**
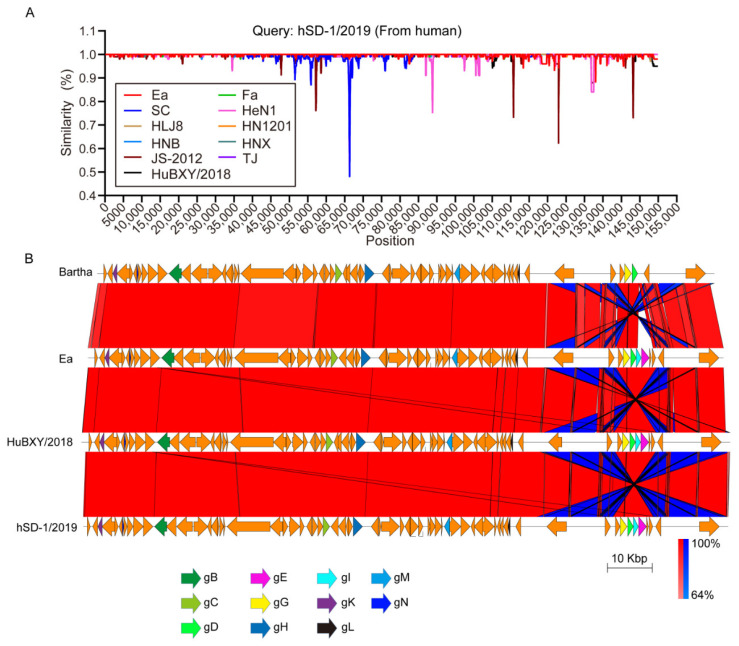
Sequence comparisons of human-originated and pig-originated PRV strains (**A**) Nucleotide similarities of the human-originated PRV strain hSD-1/2019 and the pig-originated PRV variant strains (HeN1, HLJ8, HN1201, HNB, HNX, JS-2012, TJ, and HuBXY/2018), as well as the pig-originated PRV classic strains (Ea, Fa, SC). (**B**) Comparative genomic analyses of the human-originated PRV strain hSD-1/2019, the pig-originated PRV variant strain HuBXY/2018, the pig-originated PRV strain Ea, and the vaccine strain Bartha. Color code stands for BLASTn identity of those regions between genomes. Arrows in the same colors represent putative CDSs with similar roles in different genomes.

The sequencing using PacBio technology generated a complete genome sequence 143,905 bp in length with a G+C content of 73.66% for the human-originated PRV strain hSD-1/2019. A total of 68 genes were annotated. Comparative analysis revealed that the genome sequence of the human-originated PRV genome was highly homologous to those of the pig-originated PRV strains from China (Figure 1A, Table 1). In particular, the glycoproteins-encoding genes *UL27* (encoding gB), *UL44* (encoding gC), *US6* (encoding gD), *US8* (encoding gE), *US4* (encoding gG), *UL22* (encoding gH), *US7* (encoding gI), *US3* (encoding gK), *UL1* (encoding gL), *UL10* (encoding gM), and *UL49.5* (encoding gN) harbored by the human-originated PRV strain hSD-1/2019 were also highly homologous to those of the pig-originated PRV strains from China (Figure 1B, Table 1).

### 3.2. Phylogenetic Relationship of the Human and Pig-Originated PRV Strains

**Figure 2:**
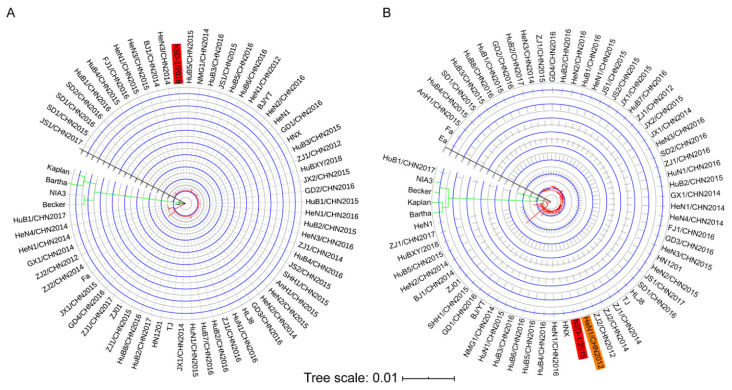
Phylogenetic analyses of human-originated and pig-originated PRV strains (**A**) A maximum likelihood tree was generated based on the full length of the gC coding gene. (**B**) A maximum likelihood tree was generated based on the complete genome sequences. Maximum likelihood trees were generated by using the Beast 2 program (version 2.6.3) through the Gamma correction for site heterogeneity and the GTR model. A bootstrap value of 1000 was also applied, and the tree was visualized by using the iTOL tool.

It has been reported that PRV strains are phylogenetically divided into two genotypes according to the *gC* gene [5]. Therefore, we first performed a phylogenetic analysis of the human- and pig-originated PRV strains. Most of the pig-originated PRV strains included in the current analysis are our previously collected variant strains from the pseudorabies (PR) outbreaks in China between 2012 and 2017 [6]. Several other variant strains from the outbreaks in China after 2011, as well as PRV strains isolated in China before 2011 and/or from other countries, are also included (Table S1 in Supplementary Materials). The phylogenetic analysis based on the *gC* gene showed that the human-originated strain, hSD-1/2019, and the swine PRV strains from China were included in one clade, while the PRV strains from the other countries, including the vaccine strain Bartha, formed another clade (Figure 2A). We also performed a phylogenetic analysis on these strains according to their genome sequences. The maximum likelihood tree also revealed that the human-originated strain hSD-1/2019 and the Chinese swine PRV strains formed a phylogenetic clade, which showed a distinct relatedness to another phylogenetic clade, which was mainly composed of the pig-originated PRV strains from the other countries, including the vaccine strain Bartha (Figure 2B).

### 3.3. Glycoproteins of the Human and Pig-Originated PRV Strains

**Figure 3:**
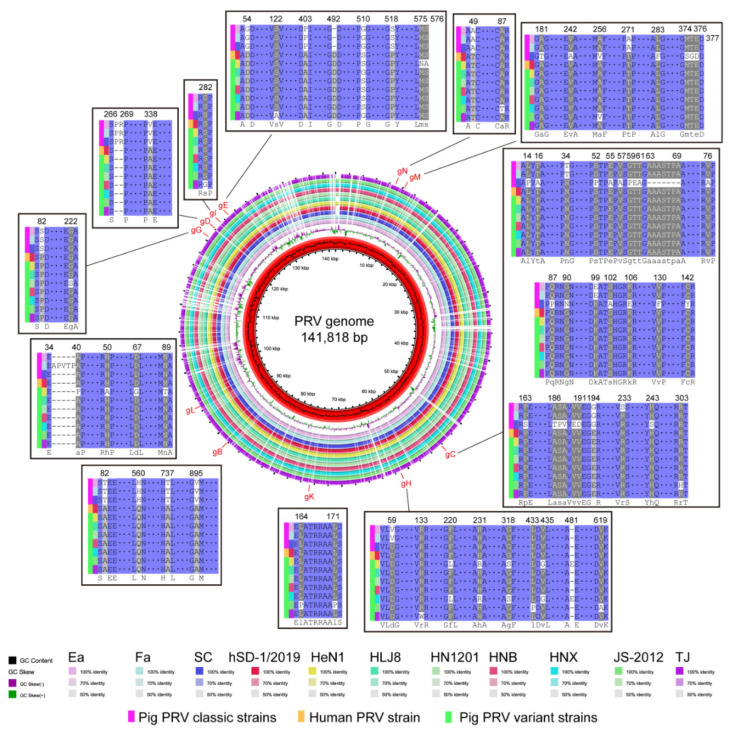
Circle map showing complete genome sequence alignments of human-originated and pig-originated PRV strains. Alignments of amino acid sequences of glycoproteins between different PRV strains were also displayed. Only sites with different amino acid residues are shown.

Glycoproteins play key roles in virus multiplication and pathogenesis [4], we, therefore, analyzed the 11 glycoproteins of the human- and pig-originated PRV strains. Overall, there were no significant differences in the amino acid components between these 11 glycoproteins of human PRV and the pig PRV variant strains (Figure 3). However, several characteristic amino acid changes were observed in glycoproteins gB, gD, gE, gG, and/or gN of the human PRV and the pig PRV variant strains compared to those of the pig PRV classic strains (Figure 3). In gB, the pig PRV classic strains possessed amino acids “T”, “H”, “T”, and “V” at sites 82, 560, 737, and 895, while the human PRV and the pig PRV variant strains all had “A”, “Q”, “A”, and “A” at these sites, respectively (Figure 3); in the gD of the human PRV and the pig PRV variant strains, the deletions of two amino acids and one amino acid change (“V→A”) occurred at sites 267, 268, and 338, compared to that of the pig PRV classic strains, respectively (Figure 3); in gE of the human PRV and the pig PRV variant strains, amino acid changes at sites 54 (“G→D”), 403 (“P→A”), 518 (“S→P”), and an insertion of one amino acid (“D”) at site 492 were observed compared to that of the pig PRV classic strains, respectively (Figure 3); in gG of the human PRV and the pig PRV variant strains, an amino acid change at site 82 (“S→P”) was observed compared to that of the classic PRV pig strains, while in the gN of the human PRV and the pig PRV variant strains, an amino acid change at site 49 (“A→T”) was observed compared to that of the classic PRV pig strains (Figure 3).

### 3.4. Diversifying Selection Analyses of the Human and Pig-Originated PRV Strains

**Figure 4:**
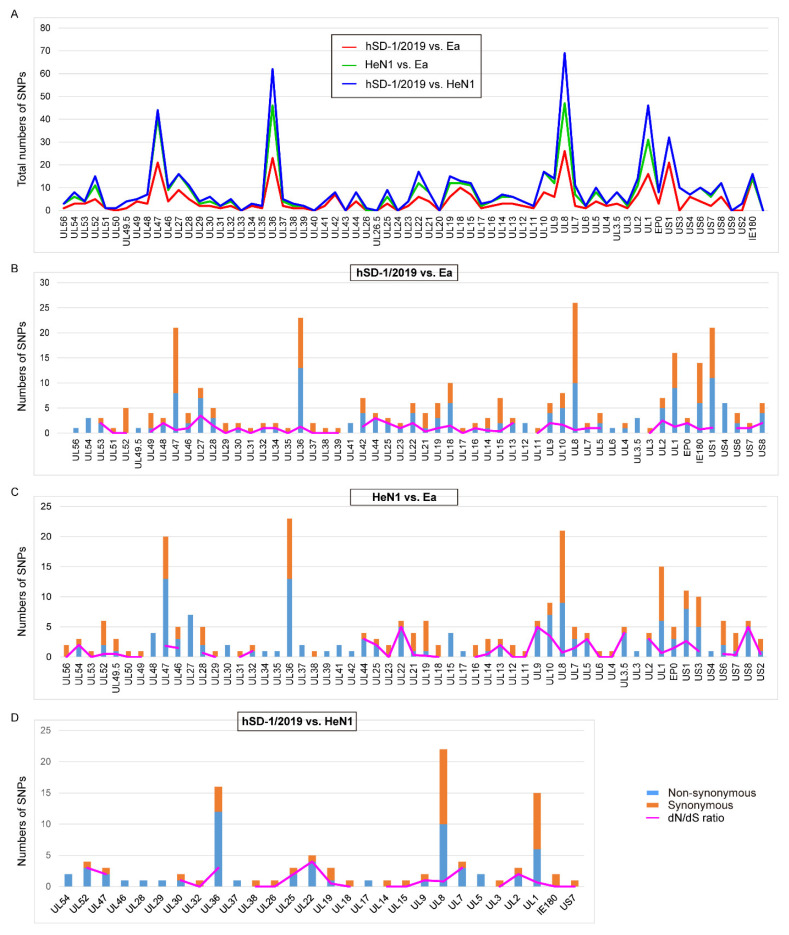
Single-nucleotide polymorphisms (SNPs) of the human- and pig-originated PRV strains (**A**) Graph showing the numbers of total SNPs identified in the complete genome sequences of human-originated PRV strain hSD-1/2019 and pig-originated PRV variant strain HeN1 compared to those of strains Ea and/or HeN1, respectively. (**B**) Graph showing the numbers of non-synonymous SNPs and synonymous SNPs, as well as the ratio of non-synonymous to synonymous substitutions (dN/dS) in the complete genome sequences of hSD-1/2019 compared to that of Ea. (**C**) Graph showing the numbers of non-synonymous SNPs and synonymous SNPs, as well as the dN/dS ratio in the complete genome sequences of HeN1 compared to that of Ea. (**D**) Graph showing the numbers of non-synonymous SNPs and synonymous SNPs, as well as the dN/dS ratio in the complete genome sequences of hSD-1/2019 compared to that of HeN1.

To explore the diversifying selection of different PRV types, the SNPs of the human-originated PRV strain hSD-1/2019, a pig PRV variant strain HeN1 (GenBank accession no. KP098534), and a classic PRV pig strain Ea (GenBank accession no. KX423960) were determined by using MUMmer software (version 3.23) [32]. A total of 508, 363, and 258 SNPs were identified in the genomes of HeN1, hSD-1/2019, and hSD-1/2019 compared to the genomes of Ea, Ea, and HeN1, respectively (Table 2). By using a local Perl command described previously [33], the coding effects of these SNPs were determined. Compared to the genome sequence of Ea, 292 SNPs determined in the genome sequence of hSD-1/2019 had coding effects, of which 153 SNPs were identified as non-synonymous SNPs and 139 SNPs were identified as synonymous SNPs (Table 2). The overall ratio between the non-synonymous to synonymous substitutions (dN/dS) of all of the coding regions of strain hSD-1/2019 compared to strain Ea was 1.10 (Table 2). In total, there were 257 SNPs with coding effects in the genome sequence of HeN1 compared to that of Ea, with 145 SNPs being identified as non-synonymous SNPs and 112 SNPs being identified as synonymous SNPs (Table 2). The dN/dS ratio between the two genome sequences was 1.29 (Table 2). Only 101 SNPs in the genome sequence of hSD-1/2019 compared to that of HeN1 were determined to have coding effects, among which 56 SNPs and 45 SNPs were identified as non-synonymous SNPs and synonymous SNPs, respectively (Table 2). The dN/dS ratio between the two genome sequences was 1.24 (Table 2). Among different comparisons (hSD-1/2019 vs. Ea; HeN1 vs. Ea; hSD-1/2019 vs. HeN1), the highest numbers of SNPs were observed in several genes such as UL47, UL36, UL8, UL1, and US1 (Figure 4). In addition, high dN/dS ratios were observed in several glycoprotein-encoding genes, such as US8, which encodes gE (Figure 4B–D).

### 3.5. Genomic Recombinant Analyses of the Human and Pig-Originated PRV Strains

**Figure 5:**
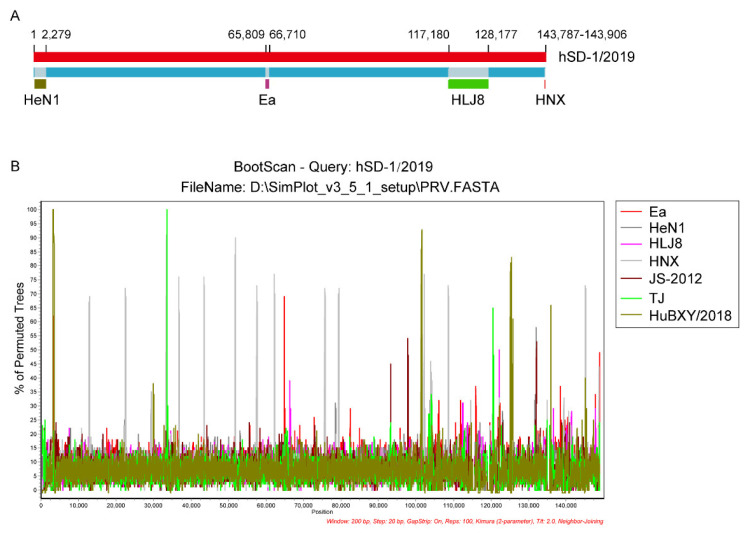
Genomic recombinant analyses of the complete genome of human-originated PRV strain hSD-1/2019 (**A**) Recombination events determined in the genome of hSD-1/2019. hSD-1/2019 genome are shown in red. The likely backbone is shown in blue. Recombination events predicted by RDP4 were shown as dark gold, purple, green, and red, respectively. Likely breakpoint positions were shown above the genome. (**B**) BootScan analysis of the complete genome sequence of hSD-1/2019. The complete genome sequence of hSD-1/2019 was used as the query sequence and compared with those of Ea, HeN1, HLJ8, HNX, JS-2012, TJ, and HuBXY/2018. The default setting of SimPlot software was used as follows: window size 200 bp, step size 20 bp.

We used the Recombination Detection Program (RDP) package Beta 4.100 [34] to determine the recombinant sequences in the default mode and a recombination event with a significance of *p* < 0.01 in at least three out of seven selected algorithms: RDP, GENECONV, BootScan, Maxchi, Chimaera, SiScan, and 3Seq, was considered to be reliable [35]. The results revealed that the human-originated PRV strain hSD-1/2019 was highly probable homologous recombinant resulting from HuBXY/2018 (GenBank accession no. MT468549), HeN1 (GenBank accession no. KP098534), Ea (GenBank accession no. KX423960), TJ (GenBank accession no. KJ789182), and HLJ8 (GenBank accession no. KT824771) (Table 3). One recombinant event appeared with a beginning breakpoint at around 1 (without gaps) and an ending breakpoint at around 2,279 (without gaps), with the major parent strain of HuBXY/2018 and a minor parent strain of HeN1, encompassing the genes *UL56* and *UL54* partially; another recombinant event appeared with a beginning breakpoint at around 65,809 (without gaps) and an ending breakpoint at around 66,710 (without gaps), with the major parent strain of HuBXY/2018 and a minor parent strain of Ea, including partial *UL21*, *UL20*, and partial *UL19*; a third recombinant event appeared with a beginning breakpoint at around 117,180 (without gaps) and an ending breakpoint at around 128,177 (without gaps), with the major parent strain of TJ and a minor parent strain of HLJ8, encompassing the genes *US3*, *US4*, *US6*, *US7*, *US8*, *US9*, and *US2* as well as partial *US1*; the last recombinant event appeared with a beginning breakpoint at around 143,787 (without gaps) and an ending breakpoint at around 143,906 (without gaps), with the major parent strain of HNX and a minor parent strain of JS-2012, encompassing parts of a repeat region (Figure 5A). BootScan analysis was performed to confirm the recombination events within the genome of hSD-1/2019 by using SimPlot software (Figure 5B).

### 3.6. Cytopathic Effects Induced by Human and Pig-Originated PRV Strains in Different Cell Lines

**Figure 6:**
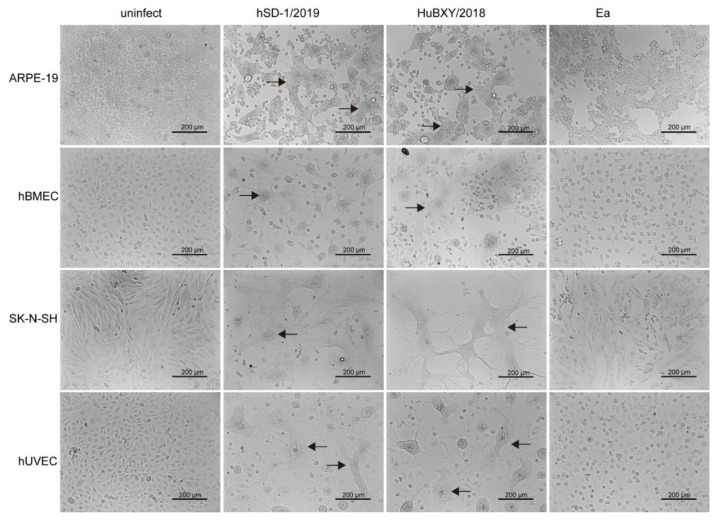
Cytopathic effects of PRV strains in different human cells. In ARPE, hBMEC, SK-N-SH, and hUVEC cells, hSD-1/2019, and HuBXY/2018 induced large syncytia, while the Ea-infected cells showed cytopathic effects characterized by single, rounded, and swollen cells. Syncytia are indicated using black arrows.

**Figure 7:**
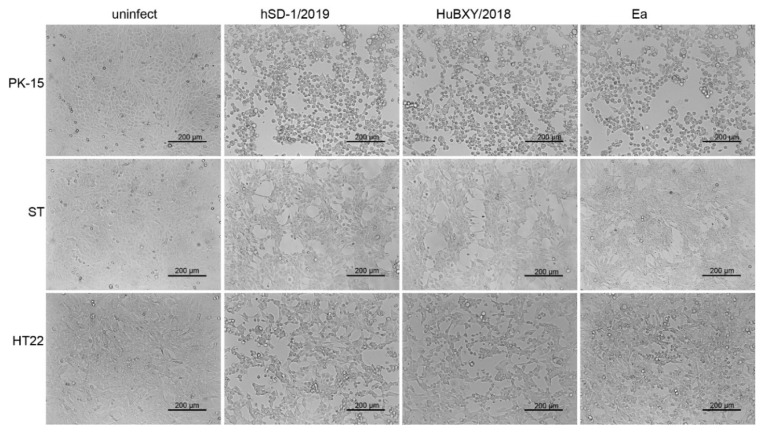
The cytopathic effects of PRV strain in swine and mouse cells. PRV strain hSD-1/2019, HuBXY/2018, and Ea induced similar cytopathic effects in PK-15, ST, and HT22 cells, respectively, which were characterized by single, rounded, and swollen cells.

The above comparative analyses revealed that the genomic characteristics of the human-originated PRV strain were more similar to those of the pig PRV variant strains rather than those of the classic PRV pig strains. Therefore, we investigated the cytopathic effects induced by the human-originated PRV strain (hSD-1/2019), pig-originated PRV variant strain (HuBXY/2018), and the classic pig-originated PRV strain (Ea) in different cell lines. Strikingly, both the human-originated PRV strain and the pig-originated variant strain formed large syncytia in the human-sourced cells (ARPE-19, hBMEC, and SK-N-SH), while the human cells treated with the classic pig-originated strain showed cytopathic effects characterized with single, rounded, and swollen cells (Figure 6). However, in the porcine cells (PK-15, ST) and murine cells (HT22), the three tested PRV strains induced similar cytopathic effects, which were characterized by single, rounded, and swollen cells (Figure 7).

## 4. Discussion

Although there has been a long documented history of suspected PRV infection in humans since 1914 [36], the virus had not been isolated from infected humans until recently [18]. As the first PRV isolates from humans, knowledge about the genomic characteristics of hSD-1/2019 and its association with pig-originated PRV strains is poorly understood. In this study, sequence comparisons revealed that the genome sequence of the human-originated PRV train hSD-1/2019 was highly homologous to (average nucleotide identity ≥ 99%) those of the PRV strains originated from pigs, including the classical strains that spread in China before 2011 (e.g., strain Ea, Fa, SC) and the variant strains isolated after 2011 (e.g., strain HuBXY/2018, HeN1, HLJ8, HN1201, HNB, HNX, JS-2012, TJ). In particular, the glycoproteins gB, gC, gD, gE, gG, gH, gI, gK, gL, gM, and gN harbored by the human-originated PRV strain hSD-1/2019 were also highly homologous to those of the pig-originated PRV strains. It is known that these glycoproteins are necessary for virus multiplication and have important roles in the pathogenesis of the virus [4]. The above findings suggested that, from the genomic level, the human-originated PRV strain exhibited similar characteristics to the pig-originated PRV strains, and the human-originated PRV might display a similar mechanism for pathogenesis.

Previously, both the *gC* gene and the whole-genome sequence were used to analyze the phylogeny of PRV strains in epidemiology studies [5,6,37], and according to the *gC* gene, the PRV strains from pigs are divided into two genotypes (genotype I and II); of which genotype II strains are mainly the PRV strains circulating in China while genotype I strains are swine PRV strains isolated from the other regions [5]. Our phylogenetic analysis based on *gC* revealed that the human-originated strain hSD-1/2019 belonged to genotype II and was closest to the epidemic swine variant strains isolated in China after 2011. A similar result was illustrated by the phylogenetic analysis using the whole-genome sequence. Additionally, from the result of the phylogenetic analysis based on the whole-genome sequence, we found that the human-originated strain hSD-1/2019 was closest related to one of our previously collected pig-originated strain HeN1/CHN2012 (GenBank accession no. MK642583) at the whole-genome level. HeN1/CHN2012, also named SMX, was isolated from neonatal piglets with severe neurological disorders, including tremble, convulsion, and opisthotonus, in May 2012 in a PR-outbreak pig farm that used commercial Bartha-K61 vaccine as a routine vaccination procedure in Henan province in China [38]. Our previous study revealed that the virus variants with defects in TK, gE, and gI from this strain protected growing pigs against the lethal challenge of PRV variant strains, while the known vaccine strain Bartha could not [38]. These findings suggest that the human-originated strain hSD-1/2019 has a very close phylogenetic relationship with the pig epidemic variant strain in China, highlighting the possibility that hSD-1/2019 is a pig-originated strain and it may transmit to humans in a certain condition.

PRV strains encode 11 glycoproteins, which are beneficial for their multiplication and pathogenesis [4]. Of particular note is gD, which mediates the binding to the host cell by using the nectin-1 receptor and, therefore, contributes to the viral entry into the host cells [39,40]. It has been shown that the gD protein from pig-originated PRV strains engages both human and swine-origin nectin-1 with similar binding affinities, and the nectin-1 proteins and those key amino acid residues required for virus binding in this protein are conserved across many different species (including pig, human, mouse, bovine species, sheep, goat, cat, dog, bat) [7,39]. The sequence alignments revealed that the gD protein of the human PRV was highly homologous (100% amino acid similarity) to that of the pig-originated PRV variant strains. The above findings might provide evidence to explain why PRV strains can infect both pigs and humans. We also determined several characteristic amino acid changes in the gD protein of the human PRV and pig-originated PRV variant strains compared to that of the pig-originated PRV classic strains. However, these changes might have no effect on the function of gD binding to nectin-1 because the binding of gD protein from a classic PRV pig strain Becker to both human and pig-origin nectin-1 proteins has been shown [39]. In addition to gD, the other glycoproteins, such as the gB of the human-originated PRV strain, were also highly homologous to those of the pig PRV variant strains. These findings may explain why hSD-1/2019 exhibits similar immunogenicity as PRV variant strains, as determined by our previous cross-neutralizing assays [18].

The analyses of the SNPs revealed high dN/dS ratios of the human-originated PRV strain and the pig-originated PRV variant strain compared to the pig-originated PRV classic strain, which suggests that the human-originated PRV strain and the pig-originated PRV variant strain are under diversifying selection, as dN/dS ratio is commonly used as a measure of purifying versus diversifying selection [41]. From the import of an inactivated vaccine derived from PRV strain Bartha in the 1970s to the circulation of pig PRV variant strains in late 2011 [9], pig herds in China had been vaccinated with Bartha-K61 or the other vaccines derived from PRV classic strains such as Ea for over 40 years. The long-time continuous and wide application of these vaccines might be an important contributor to the diversifying selection that led to the emergence of PRV variant strains. High dN/dS ratios were also found in several glycoprotein encoding genes, suggesting these genes are also under diversifying selection, which might be due to their frequent interactions with the host immune systems. Recombinant detection found several recombinant events in the genome of the human-originated PRV strain hSD-1/2019. These recombinant events resulted from several pig-originated PRV variant strains, which were isolated recently (between 2012 and 2018), and a classic PRV pig strain Ea, which was isolated in 1990. In addition, hSD-1/2019 also has a close relationship to these strains. While reports of recombination of PRV between endemic variant strains and classic strains are limited, recombination between PRV endemic strains and vaccine strains has been reported, and this recombination is speculated to be responsible for the emergence of novel strains [42]. Therefore, it may also be speculated that PRV endemic strains and earlier strains in China are probably the parental strains of human-originated PRV. These above findings may also remind us to take into consideration the recombination of different types of PRV strains during taking actions to control PRV infections in China, although the evolutionary rate of herpesvirus is very low [42].

Our cell infection experiments revealed that both human- and pig-originated PRV strains displayed a good adaption and could induce cytopathic effects in different cells from both humans, pigs, and mice. It is not a surprise to obtain these results since previous studies have found that the key receptor (nectin-1) contributing to PRV entry into the host cells is conserved across many different species, including pigs, humans, and mice [7,39], and our comparative analysis performed in this study revealed that the viral protein (gD) engaging this receptor was conserved between the human and pig-originated PRV strains. More strikingly, we found that both human PRV and pig variant PRV formed large syncytia in human cell lines (ARPE, hBMEC, and SK-N-SH); while the classic pig PRV did not. These findings indicate that both human PRV and pig variant PRV strains demonstrate a better cell-associated spread, as syncytia formation contributes to the spread of the virus between cells [43]. Although detailed mechanisms for this difference should be further explored, a recent study has found SARS-CoV-2 B.1.617 variants could also form prominent syncytia, and this capacity facilitates the furin-mediated spike cleavage and enhances and accelerates cell–cell fusion [44]. Correspondingly, the capacity of both human PRV and pig variant PRV forming syncytia may also contribute to the virus spread and immune escape. In the next step, we intend to investigate this hypothesis.

Among the different proteins of PRV, gB, gH/gL, and gK that have been demonstrated to be necessary for virus-mediated syncytia formation, while gE/gI and gM are not necessary, but they could regulate cell fusions [43]. Several previous studies showed that amino acid mutations in gB, including lacking the C-terminal 29 amino acids [45] or the mutation of the dileucine motif in the gB tail [46], were beneficial for syncytia formation and cell spread, while syncytia formation was significantly decreased in mutants deficient in gE [47]. In this study, we also found several amino acid mutations in the main glycoproteins of the human- and pig-originated variant strains compared to those of the pig-originated classic strains (Figure 3). However, it remains to be addressed whether these mutations are associated with the observation that the variant strains demonstrate better syncytia formation than the classic strains in human cell lines. Currently, the host factors associated with PRV-mediated syncytia formation has not been well revealed. It was reported that Isobavachalcone, an Akt signaling pathway inhibitor, could inhibit PRV by impairing virus-mediated cell-to-cell fusion [48]. In addition, it was stated that syncytia were formed by the fusion of adjacent cells through the binding of viral glycoproteins expressed on the infected cell membrane with the corresponding receptors expressed on adjacent uninfected cell membranes [43]. Clearly, the identification of viral glycoproteins and corresponding receptors is necessary for clarifying the mechanism of syncytial formation.

## 5. Conclusions

We reported the complete genome sequence and the genomic characteristics of the first human-originated PRV strain hSD-1/2019. Genomic comparative analyses of human-originated and pig-originated PRV strains revealed that the complete genome sequence of hSD-1/2019 was highly homologous to those of the pig PRV strains. Phylogenetic analyses revealed a very close relationship between hSD-1/2019 and PRV endemic strains in China, particularly the variant strains circulating recently. In addition, our sequence alignments found the glycoproteins important for viral multiplication and pathogenesis in hSD-1/2019 were highly similar to those of the pig endemic strains, and recombination detection suggested that hSD-1/2019 was probably the recombinant of several PRV variant strains and the earlier classic strain. The initial cell infection assays showed that both human- and pig-originated PRV strains displayed a good adaption and could induce cytopathic effects in cells from humans, pigs, and mice. Moreover, both human PRV and pig variant PRV formed large syncytia in human cell lines. These findings further expand the current understanding of the molecular basis for the pathogenesis and interspecies transmission of PRV.

## Figures and Tables

**Table 1:** Nucleotide sequence identities between hSD-1/2019 and pig PRV representative strains.

	Average Nucleotide Identity (%)											
	hSD-1/2019 (Human PRV; GenBank Accession No. MT468550)											
Type	Pig PRV Classic Strain			Pig PRV Variant Strain								Vaccine Strain
Strain	Ea	Fa	SC	HeN1	HLJ8	HN1201	HNB	HNX	JS-2012	TJ	HuBXY/2018	Bartha
GenBank accession	KX423960	KM189913	KT809429	KP098534	KT824771	KP722022	KM189914	KM189912	KP257591	KJ789182	MT468549	JF797217
Year of isolation	1990	1990	1990	2012	2014	2012	2012	2012	2012	2012	2018	1950s
Place of isolation	China	China	China	China	China	China	China	China	China	China	China	Hungry
Complete genome	99.36%	99.44%	99.03%	99.45%	99.83%	99.82%	99.83%	99.90%	99.65%	99.80%	99.62%	96.73%
*UL27*	99.82%	99.82%	99.82%	100%	100%	100%	100%	100%	100%	100%	99.96%	98.14%
*UL44*	99.66%	99.73%	95.83%	100%	100%	100%	100%	100%	99.93%	100%	100%	95.11%
*US6*	99.18%	99.18%	99.18%	100%	100%	100%	100%	100%	100%	100%	100%	98.43%
*US8*	99.41%	99.48%	99.48%	99.89%	99.94%	100.00%	100.00%	100.00%	100.00%	99.83%	100.00%	Deletion
*US4*	99.93%	99.93%	99.93%	100.00%	100.00%	99.87%	100.00%	100.00%	99.93%	100.00%	100.00%	99.13%
*UL22*	99.95%	99.95%	99.95%	99.61%	100.00%	100.00%	100.00%	100.00%	99.90%	99.95%	99.95%	99.85%
*US7*	99.82%	99.82%	99.82%	99.91%	99.91%	100.00%	99.91%	100.00%	100.00%	99.91%	100.00%	Deletion
*US3*	99.90%	99.90%	99.90%	100.00%	100.00%	100.00%	100.00%	100.00%	99.90%	100.00%	100.00%	98.71%
*UL1*	100.00%	96.91%	100.00%	96.82%	100.00%	100.00%	100.00%	100.00%	100.00%	100.00%	100.00%	95.97%
*UL10*	99.92%	99.92%	99.07%	100%	100%	100%	100%	100%	99.92%	100%	99.92%	98.73%
*UL49.5*	99.33%	99.33%	99.33%	100%	100%	100%	100%	100%	99.67%	99.67%	100%	93.81%

**Table 2:** Single nucleotide polymorphisms (SNPs) analyses of different PRV strains.

Comparisons	Non-Synonymous	Synonymous	dN/dS Ratio
hSD-1/2019 vs. Ea	153	139	1.10
HeN1 vs. Ea	145	112	1.29
hSD-1/2019 vs. HeN1	56	45	1.24

**Table 3:** Algorithms of the RDP4 package used to predict the recombination event.

Recombinant Strain	Parent Major/Minor	Recombinant Region in Alignment	Model (Average *p*-Value)						
			RDP	GENECONV	BootScan	MaxChi	Chimaera	SiScan	Phylpro
hSD-1/2019	HuBXY/HeN1	1–2279	1.73 × 10^−2^	-	-	4.35 × 10^−2^	2.4 × 10^−2^	-	3.54 × 10^−2^
	HuBXY/Ea	65,809–66,710	1.98 × 10^−8^	2.99 × 10^−8^	1.96 × 10^−9^	1.22 × 10^−2^	1.20 × 10^−2^	9.60 × 10^−9^	9.47 × 10^−6^
	TJ/HLJ8	117,180–128,177	6.74 × 10^−12^	3.21 × 10^−14^	7.85 × 10^−8^	1.62 × 10^−8^	1.18 × 10^−4^	4.28 × 10^−30^	4.02 × 10^−10^
	HNX/JS	143,787–143,906	1.69 × 10^−19^	4.29 × 10^−22^	1.23 × 10^−20^	5.84 × 10^−7^	-	3.35 × 10^−10^	-

## Data Availability

All data generated or analyzed during this study are included in this published article. The complete genome sequence of PRV strain hSD-1/2019 is deposited in NCBI. GenBank accession number is MT468550.

## Supplementary Materials

The following supporting information can be downloaded at: https://www.mdpi.com/article/10.3390/v15010170/s1, Table S1: PRV strains used for bioinformatical analyses in the present study.
