# Supplementary material for: Cytopathic and Genomic Characteristics of a Human-Originated Pseudorabies Virus
Source: Viruses. 2023 Jan 5;15(1):170. doi: 10.3390/v15010170 (PMC9862444; doi:10.3390/v15010170)
Supplement: Supplementary file 1 [file viruses-15-00170-s001.zip › viruses-2129173-Table S1.pdf]

**Table S1.** PRV strains used for bioinformatical analyses in the present study

| Strain name | GenBank accession | Country (province) |
|---|---|---|
| AnH1/CHN2015 | MK618718 | China (Anhui) |
| BJ1/CHN2014 | MK642564 | China (Beijing) |
| FJ1/CHN2016 | MK642565 | China (Fujian) |
| GD1/CHN2016 | MK642566 | China (Guangdong) |
| GD2/CHN2016 | MK642567 | China (Guangdong) |
| GD3/CHN2016 | MK642568 | China (Guangdong) |
| GD4/CHN2016 | MK642569 | China (Guangdong) |
| GX1/CHN2014 | MK642585 | China (Guangxi) |
| HeN1/CHN2012 | MK642583 | China (Henan) |
| HeN1/CHN2014 | MK642584 | China (Henan) |
| HeN2/CHN2014 | MK642582 | China (Henan) |
| HeN3/CHN2014 | MK787153 | China (Henan) |
| HeN4/CHN2014 | MK642581 | China (Henan) |
| HeN1/CHN2015 | MK642580 | China (Henan) |
| HeN2/CHN2015 | MK642579 | China (Henan) |
| HeN3/CHN2015 | MK642578 | China (Henan) |
| HeN1/CHN2016 | MK642577 | China (Henan) |
| HeN2/CHN2016 | MK642576 | China (Henan) |
| HeN3/CHN2016 | MK642575 | China (Henan) |
| HuB1/CHN2015 | MK642574 | China (Hubei) |
| HuB2/CHN2015 | MK642573 | China (Hubei) |
| HuB3/CHN2015 | MK642572 | China (Hubei) |
| HuB4/CHN2015 | MK642571 | China (Hubei) |
| HuB5/CHN2015 | MK642570 | China (Hubei) |
| HuB1/CHN2016 | MK682662 | China (Hubei) |
| HuB2/CHN2016 | MK682663 | China (Hubei) |
| HuB3/CHN2016 | MK682664 | China (Hubei) |
| HuB4/CHN2016 | MK682665 | China (Hubei) |
| HuB5/CHN2016 | MK682666 | China (Hubei) |
| HuB6/CHN2016 | MK682667 | China (Hubei) |
| HuB7/CHN2016 | MK682668 | China (Hubei) |
| HuB8/CHN2016 | MK682669 | China (Hubei) |
| HuB1/CHN2017 | MK682670 | China (Hubei) |
| HuB2/CHN2017 | MK682671 | China (Hubei) |
| HuN1/CHN2015 | MK682672 | China (Hunan) |
| HuN1/CHN2016 | MK682673 | China (Hunan) |
| JS1/CHN2015 | MK682674 | China (Jiangsu) |
| JS2/CHN2015 | MK682675 | China (Jiangsu) |
| JS1/CHN2017 | MK682676 | China (Jiangsu) |
| JX1/CHN2014 | MK787152 | China (Jiangxi) |
| JX1/CHN2015 | MK787151 | China (Jiangxi) |
| JX2/CHN2015 | MK787150 | China (Jiangxi) |

| **Strain name** | **GenBank accession** | **Country (province)** |
| --- | --- | --- |
| NMG1/CHN2014 | MK787154 | China (Iner-Mongolia) |
| SD1/CHN2015 | MK787155 | China (Shandong) |
| SD1/CHN2016 | MK787156 | China (Shandong) |
| SD2/CHN2016 | MK787164 | China (Shandong) |
| SHH1/CHN2015 | MK787156 | China (Shanghai) |
| ZJ1/CHN2012 | MK787158 | China (Zhejiang) |
| ZJ2/CHN2012 | MK787159 | China (Zhejiang) |
| ZJ1/CHN2014 | MK787160 | China (Zhejiang) |
| ZJ2/CHN2014 | MK787161 | China (Zhejiang) |
| ZJ1/CHN2015 | MK787162 | China (Zhejiang) |
| ZJ1/CHN2016 | MK787163 | China (Zhejiang) |
| ZJ1/CHN2017 | MK787149 | China (Zhejiang) |
| HN1201 | KP722022.1 | China |
| HNB | KM189914.3 | China |
| HNX | KM189912.1 | China |
| HeN1 | KP098534.1 | China |
| HLJ8 | KT824771.1 | China |
| JS-2012 | KP257591.1 | China |
| TJ | KJ789182.1 | China |
| Fa | KM189913.1 | China |
| Ea | KX423960.1 | China |
| SC | KT809429.1 | China |
| NIA3 | KU900059.1 | Northern Ireland |
| Kaplan | KJ717942.1 | United States |
| Becker | JF797219.1 | United States |
| Bartha-K61 | JF797217.1 | Hungry |
